# Commentary: Exercise-dependent BDNF as a Modulatory Factor for the Executive Processing of Individuals in Course of Cognitive Decline. A Systematic Review

**DOI:** 10.3389/fpsyg.2017.01858

**Published:** 2017-10-27

**Authors:** Felipe Stigger, Miriam A. Z. Marcolino, Rodrigo D. M. Plentz

**Affiliations:** ^1^Departament of Physiotherapy, Federal University of Health Sciences of Porto Alegre, Rio Grande do Sul, Brazil; ^2^Postgraduate Program in Rehabilitation Sciences, Federal University of Health Sciences of Porto Alegre, Porto Alegre, Brazil

**Keywords:** executive function, brain-derived neurotrophic factor, exercise, aged, cognition

The study of de Assis and de Almondes, 2017 fits the growing research movement regarding the effects of exercise over brain-derived neurotrophic factor (BDNF) and cognitive function in elderly. We read it with great interest and would like to elicit some concerning points about methodological principles applied to conduct the presented systematic review.

Systematic reviews are considered to provide the highest level of evidence for assessing the effectiveness of therapeutic interventions (Liberati et al., 2009; Oh, 2016). A systematic review, typically involves a well-defined, established, transparent and replicable method derived a priori, aiming to reduce bias through identifying, appraising, and synthesizing all potentially relevant studies on a particular topic of interest (Uman, 2011; Impellizzeri and Bizzini, 2012). Several resources are available defining methodological principles to help authors prepare and conduct a systematic review (Liberati et al., 2009; Oh, 2016).

To ensure the integrity of the design and conduction of the review, PROSPERO registration and PRISMA statement should always be used (Harris et al., 2017). Although not mandatory, registration ideally takes place before the researchers have started formal screening against inclusion criteria (Booth et al., 2012) guaranteeing that a systematic review is carefully planned and consistently conducted by the review team avoiding arbitrariness in decision-making with respect to inclusion criteria and data extraction (Shamseer et al., 2015). Although de Assis and Almondes registered their review protocol (CRD42016050017) there are discrepancies between the protocol and published review. By the end, the authors followed neither methodological description.

In our point of view, the main weaknesses of de Assis and Almondes' original article are: (1) the definition and pursuit of the research question, (2) fails to meet established eligibility criteria and (3) lack of methodological quality assessment of the included studies.

The research question distinguishes a systematic review from a narrative one and guides the search strategy and eligibility criteria. It generally contains a careful selection of PICO(S) criteria (Participants, Intervention(s), Comparator(s), Outcome(s), and Study Design) (Higgins and Green, 2011). Choosing these components provides an effective search and maximizes retrieving potential articles (Santos et al., 2007). An adequate search use internationally acknowledged keywords, and generally include several relevant electronic databases. A full search strategy description, for at least one database, enhances results reproducibility (Moher et al., 2009). However, de Assis and Almondes presented a confusing search strategy, making it difficult to replicate.

Although, de Assis and Almondes' review gives impression of inclusion of three different databases (“PubMed, Scopus and MEDLINE”), MEDLINE is the primary component of PubMed. Even accessing MEDLINE through other interface, its results are already covered in PubMed search. Also, accordingly to Cochrane, three main databases (MEDLINE, EMBASE, and CENTRAL) are recommended to find relevant studies (Chandler et al., 2013). In further analysis, to verify if potential studies were ignored in the database search choice, we adjusted their search strategy to EMBASE and CENTRAL, and found more than 1,000 records, a number more than three times higher than those pointed out by authors in their broad search. This may have led to errors in eligibility and inclusion of studies.

According to eligibility criteria, author cited that retrieved studies should “fully” meet the following inclusion criteria: aging subjects with cognitive impairments or dementia; at least one session of exercise; comparator criteria not presented; pre-post measures of BDNF and reported parameters of executive functions.

Misjudgments are noticeable considering eligibility criteria indicated above and, following the established, only Baker et al. (2010) and Nascimento et al. (2014) fully meet inclusion criteria. Tree studies do not include elderly with cognitive impairment defined as participant criteria. Ruscheweyh et al. (2011) excluded individuals with cognitive impairment and dementia (confirmed by Mini-Mental State Examination). Vaughan et al. (2014) stated as exclusion criteria individuals with cognitive impairment and dementia. And, although Anderson-Hanley et al. (2012) did not report cognitive impairment as exclusion criteria, only 38% of included participants presented mild cognitive impairment.

Inconsistencies are also related to intervention and outcome criteria. Even though exercise type was not determined by de Assis and Almondes as the intervention criteria, retrieved studies only reported aerobic training and three resistance training studies were excluded, as observed on selection process flow-chart. Moreover, measures of BDNF and executable functions must be performed pre and post exercise protocol, but Suzuki et al. (2013) reported only baseline BDNF measurement. Also, the absences of outcomes, other than executable functions, in inclusion criteria turn it difficult to confirm author's hypothesis “that increments in domains of executable functions due to the exercise regulation of BDNF in aging individuals undergoing cognitive decline depend on greater amounts of exercise-dependent BDNF stimuli than do those of memory and cognitive processing.”

As a final point, it is important to emphasize that de Assis and Almondes did not assess risk of bias of included studies. There is broad consensus among the scientific community on the relevance of assessing the methodological quality of the studies, especially when carrying out a research synthesis (Johnson et al., 2015). Problems with the design and execution of individual studies raise questions about the validity of their findings, as bias may lead them to overestimate or underestimate the true intervention effect (Higgins and Green, 2011). Tough, ignoring this process, authors did not considered the quality of the results polled in their review which could result in questionable conclusion about interventions effectiveness.

In sum, for all of the reasons stated above, we genuinely believe that it is important to point methodological issues regarding the conduction of systematic reviews aiming to improve quality of evidence synthesis that provide subsides to the evidence-based practice. Although we have outlined some major points in which we disagree in the de Assis and Almondes's review, we agree that “exercise influence on cognition is still a poorly explored field” and, even more importantly, that “further experimental research should concern better control.” Those statements provide courses on which any future research might proceed.

## Author contributions

FS contributed to the idea and writing. MM and RP contributed to the writing.

### Conflict of interest statement

The authors declare that the research was conducted in the absence of any commercial or financial relationships that could be construed as a potential conflict of interest.
